# Risk Factors for Non-O157 Shiga Toxin–Producing *Escherichia coli* Infections, United States

**DOI:** 10.3201/eid2906.221521

**Published:** 2023-06

**Authors:** Ellyn P. Marder, Zhaohui Cui, Beau B. Bruce, LaTonia Clay Richardson, Michelle M. Boyle, Paul R. Cieslak, Nicole Comstock, Sarah Lathrop, Katie Garman, Suzanne McGuire, Danyel Olson, Duc J. Vugia, Siri Wilson, Patricia M. Griffin, Carlota Medus

**Affiliations:** Centers for Disease Control and Prevention, Atlanta, Georgia, USA (E.P. Marder, Z. Cui, B.B. Bruce, L. Clay Richardson, P.M. Griffin);; Maryland Department of Health, Baltimore, Maryland, USA (M.M. Boyle);; Oregon Health Authority, Portland, Oregon, USA (P.R. Cieslak);; Colorado Department of Public Health and Environment, Denver, Colorado, USA (N. Comstock);; University of New Mexico, Albuquerque, New Mexico, USA (S. Lathrop);; Tennessee Department of Health, Nashville, Tennessee, USA (K. Garman);; New York State Department of Health, Albany, New York, USA (S. McGuire);; Connecticut Emerging Infections Program, New Haven, Connecticut, USA (D. Olson);; California Department of Public Health, Richmond, California, USA (D.J. Vugia);; Georgia Department of Public Health, Atlanta (S. Wilson);; Minnesota Department of Health, Saint Paul, Minnesota, USA (C. Medus)

**Keywords:** Escherichia coli, Shiga toxin–producing Escherichia coli, STEC, enteric infections, bacteria, case-control, foodborne illnesses, food safety, produce, meat, animals, risk, FoodNet, United States

## Abstract

Shiga toxin–producing *Escherichia coli* (STEC) causes acute diarrheal illness. To determine risk factors for non-O157 STEC infection, we enrolled 939 patients and 2,464 healthy controls in a case–control study conducted in 10 US sites. The highest population-attributable fractions for domestically acquired infections were for eating lettuce (39%), tomatoes (21%), or at a fast-food restaurant (23%). Exposures with 10%–19% population attributable fractions included eating at a table service restaurant, eating watermelon, eating chicken, pork, beef, or iceberg lettuce prepared in a restaurant, eating exotic fruit, taking acid-reducing medication, and living or working on or visiting a farm. Significant exposures with high individual-level risk (odds ratio >10) among those >1 year of age who did not travel internationally were all from farm animal environments. To markedly decrease the number of STEC-related illnesses, prevention measures should focus on decreasing contamination of produce and improving the safety of foods prepared in restaurants.

Non-O157 Shiga toxin–producing *Escherichia coli* (STEC), which encompasses all STEC serogroups other than O157, causes an estimated 219,000 US infections annually (*1*). Typical symptoms are diarrhea, abdominal cramps, and vomiting, and hemolytic uremic syndrome occurs in 1% (*2*); deaths from STEC are rare. Incidence is highest among children (*2*). Most strains isolated from US residents belong to 1 of 6 serogroups, defined by O antigens (*3**–**5*) (S. Browning, Centers for Disease Control and Prevention, December 18, 2020 email). 

Non-O157 STEC infections were underdiagnosed for decades because laboratories lacked practical detection methods (*4*,*6*–*9*). Culture-independent diagnostic tests for Shiga toxin became available in 1995. The number of laboratories using enzyme immunoassays and PCR tests to identify non-O157 STEC has been increasing since then. Reported infections increased further after non-O157 STEC infection was designated a nationally notifiable infection in 2000 (*2*,*10*).

Investigations of non-O157 STEC outbreaks have identified transmission routes, including foodborne, waterborne, from contact with animals and their environments, and person-to-person contact (*11*,*12*). Because little is known about risk factors for sporadic infections, the Foodborne Diseases Active Surveillance Network (FoodNet) conducted a large, multisite, case–control study to identify risks for sporadic non-O157 STEC infections in the United States. Centers for Disease Control and Prevention (CDC) and FoodNet site institutional review boards approved the study protocol. We obtained verbal consent from all persons ≥18 years of age and parents or legal guardians of children <18 years of age and verbal assent (in addition to parent or guardian consent) from children 12–17 years of age. 

## Methods

During 2012–2015, FoodNet conducted active, population-based surveillance for laboratory-diagnosed STEC infections in 10 sites, covering an estimated 49 million persons (15% of the US population in 2014). The catchment area included Connecticut, Georgia, Maryland, Minnesota, New Mexico, Oregon, and Tennessee and selected counties in California, Colorado, and New York. We recruited patients from each site for a consecutive 36-month period during July 1, 2012–September 1, 2015. We defined a case as isolation of non-O157 STEC from a clinical specimen of an ill person residing in a FoodNet site. We excluded cases in which a pathogen other than non-O157 STEC was detected in a non-O157 STEC–positive specimen, or the patient was lost to follow-up, did not speak English or Spanish, was part of an outbreak (except for the index patient in each site), or was not the first case in their household. We attempted to enroll 3 controls per case, matched on county and stratified by age groups: 0–1, 2–5, 6–17, 18–39, 40–59, or ≥60 years. We selected controls in all except the youngest age group from commercially available lists of residential telephone numbers, by county, that included age ranges. We selected controls <2 years of age from birth registries. We enrolled controls within 60 days after the matched case-patient’s specimen collection date. We excluded controls who did not speak English or Spanish.

We interviewed patients and controls or their guardians by telephone using a standard questionnaire that covered 385 variables and had sections on health, travel, water, animals, foods, and demographics. Most exposures, including international travel, were for the 7 days before illness began; controls were asked about exposures during the same period as case-patients. The questionnaire defined fast-food restaurants as places where food is ordered and paid for at a counter or drive-through and table-service restaurants as all sit-down or table-service restaurants. 

Clinical laboratories submitted specimens that had Shiga toxin (determined by immunoassay) or Shiga toxin genes (determined by PCR) to state public health laboratories. State public health laboratory staff identified non-O157 specimens and submitted them to CDC for serologic testing to determine O and H antigens. CDC used whole-genome sequencing to confirm the absence of O157 genes on rough isolates. 

We included all enrolled participants in descriptive analyses. International travel was examined in univariable analysis. Those reporting international travel were excluded from other risk factor analyses, which were conducted separately for infants <1 and persons >1 years of age. To control for confounding in the main risk factor analysis, we rematched controls with cases using the nearest-neighbors approach (*13*). For a given exposure, we calculated Gower distance on the basis of age, sex, state, and all exposures except the one under consideration (*14*). Using logistic regression, we established an overall threshold for Gower distance at which it was more likely that a matched control was a patient’s nearest neighbor than a randomly selected control. We matched up to 20 controls within the Gower distance with the nearest case-patient and ensured that each control was matched to only 1 case-patient. Of note, distance between 85% of patient–control pairs matched during recruitment exceeded that threshold. We excluded case-patients without matches within the threshold from the analysis for the exposure under consideration. After rematching patients with controls, information was available for patients for all but 5 exposures in at least 92% of cases: municipal water away from home (89%), private well water away from home (85%), spring water away from home (85%), prepackaged iceberg lettuce (84%), and prepackaged romaine lettuce (87%). Information was available for all except 4 exposures for at least 92% of controls: municipal water away from home (91%), contact with someone with diarrheal illness (90%), private well water away from home (82%), and spring water away from home (81%). We did not conduct imputation because results were unlikely to be affected by the low rates of missing data. 

For our analyses, we calculated odds ratios (ORs) and population attributable fractions (PAFs) to identify both individual risk and percentages at which illnesses in the population could be decreased. Because prevalence of some exposures was low among case-patients, controls, or both, we applied Firth bias-reduced penalized-likelihood logistic regression to estimate ORs and 95% CIs for each exposure, after adjusting for the matched strata generated by the nearest-neighbors approach. We calculated and adjusted p values for multiple testing using the Benjamini-Yekutieli method (*15*). We considered associations statistically significant if adjusted p was <0.05 and 95% CIs did not include 1.0. We calculated PAF using a method described elsewhere (*16*) and calculated 95% CIs for PAFs using the 95% confidence limits of ORs. We did not assess the overall statistical significance of our logistic regression models because each included only the exposure under consideration and the strata of matched case-control pairs (Appendix Table 2). 

## Results 

We identified 1,988 non-O157 STEC case-patients and 2,464 controls meeting inclusion criteria; we excluded 324 of the case-patients according to exclusion criteria (Figure). Of the 1,644 eligible patients remaining, 407 could not be reached and 318 refused to participate, leaving 939 (56.4%) total cases in the study. Nine serogroups accounted for 83% of isolates from enrolled case-patients: O26 (263, 28%), O103 (216, 23%), O111 (135, 14%), O121 (46, 5%), O118 (37, 4%), O186 (23, 2%), O5 (22, 2%), O145 (21, 2%), and O45 (21, 2%) (Table 1). The remainder of the results is limited to enrolled case-patients.

**Figure:**
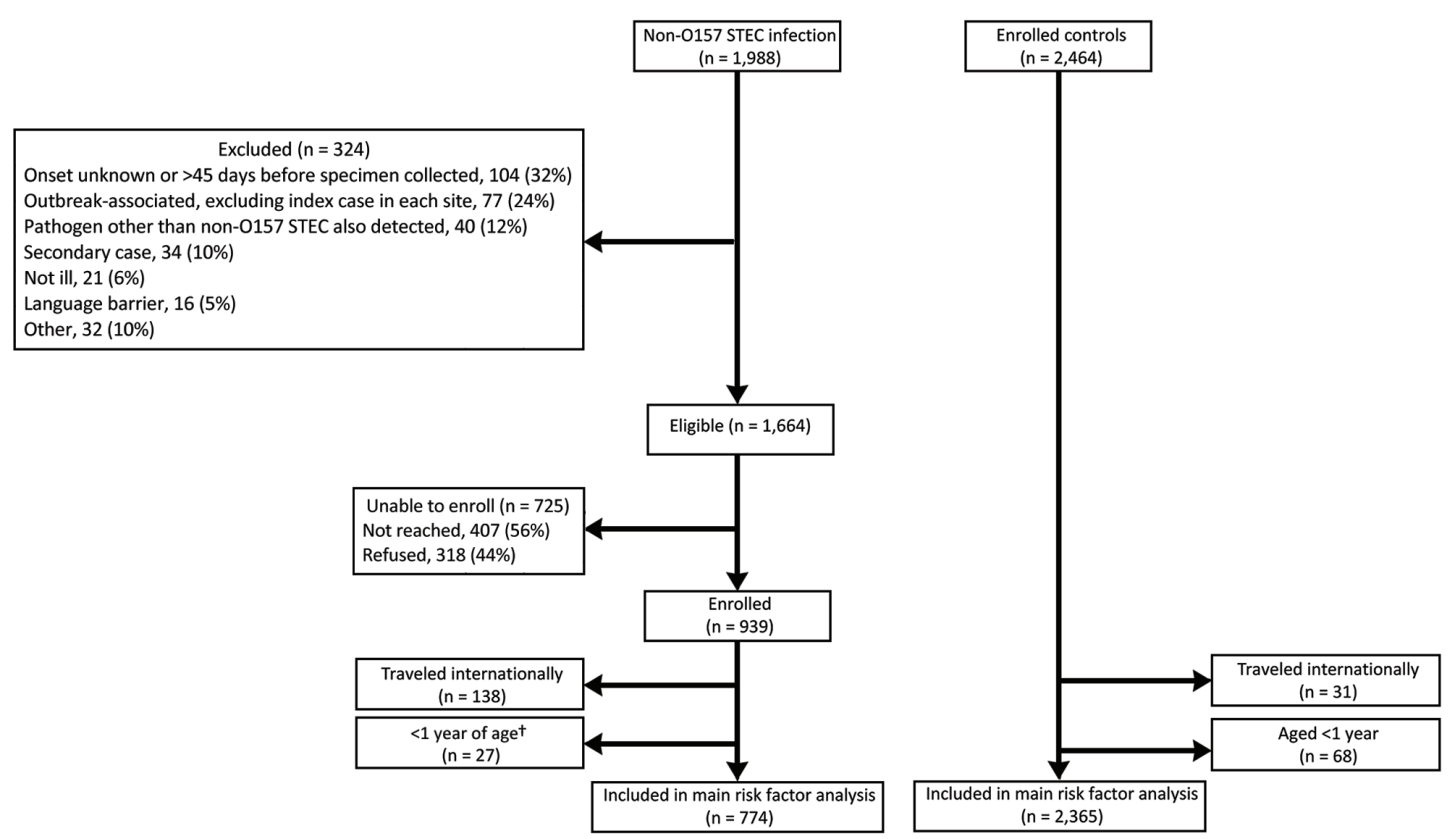
Flowchart for inclusion/exclusion in study of risk factors for non-O157 STEC infections, United States. *Campylobacter, n = 11; *Salmonella*, n = 8; *Cryptosporidium*, n = 7; STEC O157, n = 7; *C. difficile*, n = 2; *Giardia*, n = 2; *Cryptosporidium* and *Giardia*, n = 1; norovirus, n = 1; *Shigella*, n = 1. †An additional 3 infants who traveled internationally were included in the Traveled internationally box above. STEC, Shiga toxin–producing *Escherichia coli*.

**Table 1:** Demographics, clinical characteristics, and international travel among case-patients with non-O157 Shiga toxin–producing *Escherichia coli* infection, by serogroup, FoodNet case–control study, United States, 2012–2015 (n = 939)*

Category	Serogroup										
	O26	O103	O111	O121	O118	O186	O5	O145	O45	Other†	Total‡
Demographics											
Median age, y (IQR)	18 (4–33)	20 (6.5–36)	14.5 (4–27)	22.5 (8–42)	2 (1–16)	15 (3–28)	17 (4–27)	15 (5–27)	30 (17–52)	26.5 (10–62)	19 (5–36)
Sex											
F	153/263 (58)	142/216 (66)	78/134 (58)	27/46 (59)	19/37 (51)	20/32 (63)	9/22 (41)	13/21 (62)	14/21 (67)	57/98 (58)	553/939 (59)
M	110/263 (42)	74/216 (34)	56/134 (42)	19/46 (41)	18/37 (49)	12/32 (37)	13/22 (59)	8/21 (38)	7/21 (33)	41/98 (42)	341/939 (41)
Race											
White	231/251 (92)	175/196 (89)	113/126 (90)	45/45 (100)	23/29 (79)	20/21 (95)	18/20 (90)	20/21 (95)	20/20 (95)	76/86 (88)	787/868 (91)
Black	10/251 (4)	11/196 (6)	8/126 (6)	0/45 (0)	3/29 (10)	0/23 (0)	1/20 (5)	1/21 (5)	0/20 (0)	4/86 (5)	41/868 (5)
Asian	3/251 (1)	1/196 (1)	2/126 (2)	0/45 (0)	2/29 (7)	1/21 (5)	1/20 (5)	0/21 (0)	0/20 (0)	4/86 (5)	17/868 (2)
Ethnicity											
Hispanic	38/260 (15)	33/211 (16)	25/132 (19)	5/45 (11)	19/37 (51)	6/23 (26)	2/22 (9)	1/21 (5)	1/21 (5)	15/96 (16)	155/926 (17)
Clinical characteristics											
Diarrhea	260/263 (99)	214/216 (99)	133/134 (99)	45/46 (98)	37/37 (100)	23/23 (100)	21/22 (95)	21/21 (100)	21/21 (100)	97/98 (99)	929/939 (99)
Median duration, d (IQR)	7 (5–10)	7 (5–10)	6.5 (5–10)	5.5 (4–7)	8 (6.5–14)	7 (5–10)	6 (4.5–7)	7 (5–10)	7.5 (5–12)	7 (4–10)	7 (5–10)
Abdominal pain	225/254 (89)	188/206 (91)	113/123 (92)	43/43 (100)	25/31 (81)	20/23 (87)	19/21 (90)	16/18 (89)	20/20 (100)	76/96 (79)	790/889 (89)
Fatigue	175/256 (68)	149/209 (71)	92/125 (74)	33/45 (73)	20/37 (54)	17/22 (77)	15/21 (71)	16/20 (80)	15/19 (79)	65/92 (70)	636/901 (71)
Bloody feces	166/258 (64)	130/210 (62)	66/132 (50)	36/46 (78)	15/37 (41)	11/21 (52)	16/22 (73)	10/21 (48)	16/21 (76)	42/95 (44)	533/919 (58)
Nausea	124/252 (49)	128/203 (63)	69/128 (54)	20/45 (44)	12/34 (35)	9/21 (43)	5/22 (23)	13/20 (65)	12/20 (60)	47/95 (49)	474/893 (53)
Fever	99/253 (39)	86/201 (43)	58/127 (46)	12/41 (29)	16/36 (44)	8/23 (35)	5/21 (24)	10/21 (48)	6/21 (29)	26/95 (27)	343/894 (38)
Chills	82/251 (33)	93/207 (45)	49/129 (38)	14/45 (31)	9/32 (28)	5/21 (24)	6/22 (27)	5/20 (25)	8/20 (40)	31/96 (32)	323/897 (36)
Achy joints or muscles	75/242 (31)	63/197 (32)	37/117 (32)	12/41 (29)	5/28 (18)	7/22 (32)	6/20 (30)	5/19 (26)	8/20 (40)	29/90 (32)	269/847 (32)
Vomiting	65/261 (25)	68/214 (32)	41/133 (31)	14/46 (30)	9/36 (25)	5/23 (22)	9/22 (41)	10/21 (48)	4/21 (19)	29/97 (30)	273/930 (29)
HUS	1/263 (0)	0/216 (0)	1/133 (1)	0/45 (0)	0/37 (0)	0/23 (0)	0/22 (0)	4/21 (19)	0/21 (0)	2/98 (2)	8/937 (1)
Hospitalized	34/258 (13)	25/215 (12)	33/133 (25)	15/46 (33)	1/36 (3)	2/23 (9)	8/22 (36)	7/21 (33)	8/21 (38)	14/97 (14)	154/927 (17)
International travel	28/263 (11)	29/216 (13)	27/134 (20)	0/46 (0)	11/37 (30)	11/23 (48)	1/22 (5)	0/21 (0)	1/21 (5)	21/98 (21)	138/939 (15)

*Values are no. positive/no. for whom data were available (%) except as indicated. HUS, hemolytic uremic syndrome; IQR, interquartile range. †Includes O71 (n = 18), O69 (13), O165 (6), O76 (6), O177 (5), O113 (4), O50 (4), O84 (3), O91 (3), O146 (2), O156 (2), O174 (2), O178 (2), O181 (2), O22 (2), O32 (2), O77 (2), and 1 each of O1, O11, O101, O117, O123, O128, O128abc, O159, O168, O172, O175, O185, O36, O55, O65, O74, O79, O80, O82, and O98. ‡Includes infections caused by undetermined serogroups (n = 25), O rough isolates (i.e., isolates that do not express the O antigen) (11), unknown serogroup (11), and infections with >1 serogroup isolated (10). §Within 7 d before illness onset.

Nearly all patients (99%) reported diarrhea (median duration 7 days, interquartile range 5–10 days) (Table 1). Other common signs and symptoms were abdominal pain (89%), fatigue (71%), bloody feces (58%), and nausea (53%). Seventeen percent of patients were hospitalized, and 8 (1%) had hemolytic uremic syndrome develop. 

International travel was significantly associated with infection in univariable analysis; 138/939 (15%) patients reported international travel, compared with 31/2,464 (1%) controls (matched OR 14.2, 95% CI 9.0–23.3) (Table 1). The most common destination among patients traveling internationally was Mexico (68, 49%). The rank order of non-O157 STEC serotypes among international travelers was similar to that for domestic cases except for the absence of O121. O186 (11/23, 48%) and O118 (11/37, 30%) were the serogroups with the highest percentages of patients who had recently traveled internationally. 

Most patients (801/939) and controls (2,433/2,464), including 27 infant case-patients and 68 infant controls, had not recently traveled internationally. Patient median age was 18 years (interquartile range 4–35 years); 57% were female, 90% White, and 17% of Hispanic ethnicity (Table 2). Median age was significantly lower for patients (18 years) than for controls (22 years). Patients were also more likely than controls to be White (90% vs. 87%) and of Hispanic ethnicity (17% vs. 10%) and less likely to be Black (5% vs. 7%). Among FoodNet sites, the most cases were in Minnesota (226, 28%), followed by Tennessee (107, 13%), Oregon (91, 11%), Georgia (88, 11%), California (61, 8%), New York (58, 7%), Colorado (54, 7%), Connecticut (46, 6%), New Mexico (40, 5%), and Maryland (30, 4%). International travel was the only factor significantly associated with infection among 3/30 (10%) infants, compared with none among 68 controls (OR 32.8, 95% CI 1.5–4,607.2). No food, environmental, water, or other exposure we examined among infants who had not traveled internationally was significantly associated with illness (Appendix Table 1). 

**Table 2:** Demographic characteristics of case-patients with non-O157 Shiga toxin–producing *Escherichia coli* infection and controls without international travel, FoodNet case–control study, United States, 2012–2015*

Characteristic	Case-patients, n = 801	Controls, n = 2,433
Age, y median (IQR)	18 (4–35)	22 (6–39)
Sex		
F	457/801 (57)	1,425/2,410 (59)
M	344/801 (43)	982/2,410 (41)
Race		
White	667/739 (90)	2,016/2,310 (87)
Black	35/739 (5)	167/2,310 (7)
Asian	15/739 (2)	46/2,310 (2)
Ethnicity		
Hispanic	133/789 (17)	236/2,399 (10)†

*Values are no. positive/no. for whom data were available (%) except as indicated. †p<0.05 compared with case-patients.

Among persons >1 year of age who had not traveled internationally, significant PAFs (>20%) were largest for eating lettuce (PAF 39.3%; OR 2.6), tomatoes (PAF 21.3%; OR 1.7), or at a fast-food restaurant (PAF 22.5%; OR 1.7) (Table 3). Other produce exposures with high PAFs (10%–19%) were eating watermelon (PAF 19.0%; OR 2.4), including prepared inside the home (PAF 10.9%; OR 1.7); eating tomatoes prepared in a restaurant (PAF 13.7%; OR 2.5); eating exotic fruit, such as kiwi, avocado, or mango (PAF 13.2%; OR 1.7); and eating iceberg lettuce prepared in a restaurant (PAF 12.9%; OR 2.7). The highest ORs among fruit and vegetable exposures were for raspberries (PAF 2.2%; OR 7.7), cantaloupe (PAF 3.2%; OR 4.3), exotic fruit (PAF 5.8%; OR 3.9), and pineapple (PAF 3.8%; OR 3.6) prepared in a restaurant. However, <8% of patients had exposure to any 1 of those. 

**Table 3:** Risk factors associated with domestically acquired non-O157 Shiga toxin-producing *Escherichia coli* infection, FoodNet case–control study, United States, 2012–2015*

Consumption/exposure*†	Cases,‡ n = 774	Controls,‡ n = 2,365	Multivariable analysis		
			OR (95% CI)	PAF (95% CI)	p value§
Fruits and vegetables					
Lettuce	288/447 (64)	710/1,268 (56)	2.6 (1.8–3.6)	39.3 (29.2–46.6)	<0.001
Tomatoes	227/435 (52)	605/1,265 (48)	1.7 (1.3–2.3)	21.3 (10.4–29.4)	0.03
Prepared outside the home	101/443 (23)	175/1,266 (14)	2.5 (1.8–3.5)	13.7 (10.0–16.3)	<0.001
Watermelon	145/448 (32)	302/1,276 (24)	2.4 (1.8–3.4)	19.0 (14.0–22.7)	<0.001
Prepared inside the home	120/445 (27)	291/1,274 (23)	1.7 (1.2–2.3)	10.9 (5.4–15.0)	0.03
Exotic fruit such as kiwi, avocado	140/444 (32)	336/1,274 (26)	1.7 (1.3–2.3)	13.2 (6.7–18.0)	0.02
Prepared outside the home	35/449 (8)	30/1,275 (2)	3.9 (2.1–7.1)	5.8 (4.2–6.7)	<0.001
Iceberg lettuce prepared outside the home	86/415 (21)	156/1,245 (13)	2.7 (1.8–3.9)	12.9 (9.4–15.4)	<0.001
Pineapple	98/449 (22)	221/1,279 (17)	1.8 (1.3–2.6)	9.9 (5.0–13.3)	0.02
Prepared outside the home	23/440 (5)	29/1,274 (2)	3.6 (1.8–7.1)	3.8 (2.3–4.5)	0.02
Salsa	71/442 (16)	137/1,275 (11)	1.9 (1.3–2.8)	7.6 (3.7–10.3)	0.04
Onions other than white or red prepared outside the home	54/438 (12)	87/1,260 (7)	2.5 (1.6–4.0)	7.4 (4.6–9.2)	0.006
Romaine lettuce prepared outside the home	51/431 (12)	95/1,227 (8)	2.2 (1.4–3.4)	6.4 (3.4–8.3)	0.03
Peppers prepared outside the home	41/440 (9)	56/1,274 (4)	3.0 (1.8–5.0)	6.2 (4.2–7.5)	0.002
Raw spinach prepared outside the home	30/445 (7)	44/1,286 (3)	2.9 (1.7–5.2)	4.4 (2.7–5.4)	0.02
Cantaloupe prepared outside the home	18/431 (4)	22/1,274 (2)	4.3 (1.9–9.9)	3.2 (2.0–3.8)	0.02
Raspberries prepared outside the home	11/432 (3)	10/1,270 (1)	7.7 (2.4–27.5)	2.2 (1.5–2.5)	0.03
Other					
Eat at a fast-food restaurant	245/434 (56)	580/1,278 (45)	1.7 (1.3–2.2)	22.5 (11.7–30.7)	0.02
Eat at a table-service restaurant	207/454 (46)	425/1,276 (33)	1.7 (1.3–2.3)	19.4 (11.5–25.4)	0.002
Stomach acid–reducing medications in 4 weeks before illness	93/430 (22)	174/1,261 (14)	2.1 (1.5–2.9)	11.3 (7.3–14.2)	<0.001
Contact with someone with diarrheal illness	56/408 (14)	55/1,153 (5)	3.6 (2.2–5.7)	9.9 (7.6–11.3)	<0.001
Meat, poultry, pork, and seafood					
Chicken prepared outside the home	203/446 (46)	465/1,269 (37)	1.6 (1.2–2.0)	16.3 (7.6–23.1)	0.04
Pork prepared outside the home	71/450 (16)	86/1,276 (7)	2.9 (1.9–4.2)	10.2 (7.6–12.0)	<0.001
Beef prepared at table service restaurant	85/443 (19)	135/1,258 (11)	2.1 (1.5–3.0)	10.1 (6.2–12.9)	0.003
Ground beef hamburger prepared at a table service restaurant	44/445 (10)	73/1,276 (6)	2.4 (1.5–3.8)	5.8 (3.4–7.3)	0.01
Pink ground beef hamburger prepared at a table service restaurant	17/443 (4)	7/1,280 (1)	9.0 (3.5–24.7)	3.4 (2.7–3.7)	<0.001
Environmental					
Live on, visit, or work on a farm, petting zoo, or fair	72/430 (17)	61/1,258 (5)	8.0(4.7–14.1)	14.7 (13.1–15.6)	<0.001
With cows or calves present	42/426 (10)	29/1,258 (2)	9.3 (4.7–19.2)	8.8 (7.8–9.3)	<0.001
With cows present	40/429 (9)	29/1,257 (2)	6.8 (3.5–13.5)	8.0 (6.7–8.6)	<0.001
With chickens present	27/430 (6)	9/1,255 (1)	26.1 (9.1–87.2)	6.0 (5.6–6.2)	<0.001
With calves present	21/424 (5)	9/1,250 (1)	23.3 (7.4–88.9)	4.7 (4.3–4.9)	<0.001
With goats present	21/435 (5)	8/1,250 (1)	15.7 (5.3–52.8)	4.5 (3.9–4.7)	<0.001
And have contact with cows or calves	19/423 (4)	6/1,246 (0)	18.8 (5.8–70.6)	4.3 (3.7–4.4)	<0.001
With sheep present	14/430 (3)	7/1,249 (1)	13.2 (3.9–51.4)	3.0 (2.4–3.2)	0.002
And have contact with cows	13/435 (3)	7/1,244 (1)	8.7 (2.7–32.9)	2.6 (1.9–2.9)	0.01
With pigs present	12/429 (3)	6/1,252 (0)	13.6 (3.5–65.0)	2.6 (2.0–2.8)	0.008
And have contact with calves	9/430 (2)	4/1,242 (0)	11.8 (2.9–59.0)	1.9 (1.4–2.1)	0.02
Visit a farm	40/434 (9)	25/1,271 (2)	9.0 (4.6–17.9)	8.2 (7.2–8.7)	<0.001
With cows present	21/436 (5)	9/1,262 (1)	7.7 (3.2–19.5)	4.2 (3.3–4.6)	<0.001
With chickens present	12/430 (3)	5/1,270 (0)	10.0 (3.4–31.2)	2.5 (2.0–2.7)	0.002
With horses present	12/434 (3)	5/1,269 (0)	12.8 (4.0–46.6)	2.5 (2.1–2.7)	<0.001
With calves present	10/436 (2)	5/1,264 (0)	9.4 (2.6–38.9)	2.0 (1.4–2.2)	0.03
With sheep present	7/427 (2)	1/1,262 (0)	19.7 (3.7–138.7)	1.6 (1.2–1.6)	0.02
And have contact with horse feed	5/438 (1)	2/1,269 (0)	20.9 (3.6–185.6)	1.1 (0.8–1.1)	0.03
Household member visited/worked on farm with animals	43/435 (10)	49/1,254 (4)	3.5 (2.1–5.9)	7.1 (5.1–8.2)	<0.001
Live on a farm	28/444 (6)	23/1,277 (2)	5.6 (2.6–12.2)	5.2 (3.9–5.8)	<0.001
With chickens present	11/443 (2)	2/1,274 (0)	28.1 (6.5–178.6)	2.4 (2.1–2.5)	<0.001
With pigs present	3/443 (1)	0/1,274 (0)	66.9 (4.7–9,270.2)	0.7 (0.5–0.7)	0.05
Camping	32/441 (7)	31/1,268 (2)	3.2 (1.8–5.7)	5.0 (3.2–6.0)	0.006
Household member visited/worked on farm with cows	22/431 (5)	19/1,248 (2)	5.1 (2.4–11.3)	4.1 (3.0–4.7)	0.002
Contact with wild deer or elk or their droppings	20/406 (5)	13/1,214 (1)	4.7 (2.2–10.4)	3.9 (2.7–4.5)	0.006
Contact with goats	9/438 (2)	3/1,255 (0)	21.2 (4.3–145.7)	2.0 (1.6–2.0)	0.005
Water					
Swim or play in lake, river, or stream	41/432 (9)	53/1,249 (4)	2.5 (1.5–4.2)	5.7 (3.2–7.2)	0.02
Swallow water from lake	30/433 (7)	35/1,238 (3)	3.0 (1.7–5.3)	4.6 (2.7–5.6)	0.02
Drink untreated water such as lake, spring, river	18/434 (4)	9/1,252 (1)	6.6 (2.8–16.1)	3.5 (2.7–3.9)	0.001

*Values are no. exposures/no. for whom data were available (%) except as indicated. OR, odds ratio; PAF, population attributable fractions. †In the 7 d before illness began unless otherwise specified. Interviewers told respondents to consider foods prepared at any home to be prepared at home and foods prepared at a restaurant or commercial food service establishment to be prepared outside the home. ‡The initial sample for each exposure was 774 noninfant nontraveler cases and 2,365 noninfant nontraveler controls. During nearest-neighbors matching, cases and controls without a match were excluded for the exposure under consideration. Thus, the numbers of cases and controls that were matched and included in the analysis of each exposure is smaller than the total. §Adjusted for multiple testing using the Benjamini-Hochberg-Yekutieli method.

Eating at a table service restaurant also had a high PAF (19.4%; OR 1.7). Of the 24 food-related risk factors identified, 17 were related to preparation in a restaurant and 1 to preparation inside the home; the other 6 did not specify a place of preparation. Meats with significant high PAFs (10%–19%) were chicken prepared in a restaurant (PAF 16.3%; OR 1.6), pork prepared in a restaurant (PAF 10.2%; OR 2.9), and beef prepared at a table-service restaurant (PAF 10.1%; OR 2.1). The highest OR among meat and seafood products was for eating pink hamburger from a table-service restaurant (PAF 3.4%; OR 9.0). Eating ground beef hamburger (PAF 5.8%; OR 2.4) at a table-service restaurant was also a significant risk factor. However, 9 of 21 factors significantly associated with lower risk of illness were related to beef (Appendix Table 2). 

Although living or working on or visiting a farm, petting zoo, or fair (PAF 14.7%; OR 8.0) was the only significant environmental exposure with a PAF ≥10%, many significant animal environment-associated exposures had ORs >10. Those included exposures to calves, chickens, cows, goats, horses, pigs, and sheep. Taking stomach acid-reducing medications in the 4 weeks before illness (PAF 11.3%; OR 2.1) was the only other significant risk factor with PAF ≥10% or OR >10. 

Among the 5 risk factors for STEC O26 infection, only 1, contact with someone with diarrheal illness (PAF 10.8%, OR 5.7), had a PAF ≥10%; the other 4, all with ORs ≥10, were animal environment exposures. Among the 7 risk factors associated with STEC O103 infection, 3 had PAFs ≥10% and the other 4 had ORs >14. The highest PAFs were for living or working on, or visiting a farm, petting zoo, or fair (PAF 22.0%, OR 7.2) and for eating iceberg lettuce in a restaurant (PAF 20.1; OR 4.5). One risk factor was identified for STEC O111: living or working on, or visiting a farm, petting zoo, or fair (PAF 20.3%; OR 15.4) (Table 4). 

**Table 4:** Risk factors associated with domestically acquired non-O157 Shiga toxin–producing *Escherichia coli* infections by serogroup, FoodNet case–control study, United States, 2012–2015*

Serogroup and exposure†	Case-patients	Controls	Multivariable analysis		
			OR (95% CI)	PAF (95% CI)	p value§
O26, n = 231					
Contact with someone with diarrheal illness	16/122 (13)	11/370 (3)	5.7 (2.4–14.4)	10.8 (7.6–12.2)	0.04
Environmental					
Live or work on, or visit a farm, petting zoo, or fair					
With chickens present	11/143 (8)	1/410 (0)	35.5 (6.9–319.6)	7.5 (6.6–7.7)	0.003
With cows present	11/140 (8)	4/399 (1)	13.6 (3.6–62.0)	7.3 (5.7–7.7)	0.04
With cows or calves present	11/141 (8)	5/394 (1)	13.7 (3.5–65.5)	7.2 (5.6–7.7)	0.04
Visit a farm with chickens present	7/139 (5)	1/421 (0)	24.3 (4.7–172.0)	4.8 (4.0–5.0)	0.04
O103, n = 179					
Environmental					
Live or work on, or visit a farm, petting zoo, or fair	24/94 (26)	22/315 (7)	7.2(2.9–19.4)	22.0 (16.6–24.2)	0.008
With cows or calves present	12/95 (13)	6/334 (2)	24.9 (5.3–169.3)	12.1 (10.2–12.6)	0.008
With calves present	7/97 (7)	2/330 (1)	60.8 (6.7–2,615.0)	7.1 (6.1–7.2)	0.02
Live on a farm	11/101 (11)	5/328 (2)	15.8 (3.8–77.8)	10.2 (8.1–10.8)	0.02
Contact with wild deer or elk or their droppings	9/98 (9)	2/327 (1)	14.6 (3.7–69.1)	8.6 (6.7–9.1)	0.02
Visit a farm with horses present	5/93 (5)	1/316 (0)	60.1 (6.4–5,983.0)	5.3 (4.5–5.4)	0.02
Fruits and vegetables					
Iceberg lettuce prepared outside the home	24/93 (26)	37/290 (13)	4.5 (2.1–9.9)	20.1 (13.7–23.2)	0.02
O111, n = 104					
Environmental					
Live on, visit, or work on a farm, petting zoo, or fair	13/60 (22)	11/190 (6)	15.4 (4.1–73.9)	20.3 (16.3–21.4)	0.03

*Values are no. exposures/no. for whom data were available (%) except as indicated. OR, odds ratio; PAF, population attributable fractions. †In the 7 d before illness unless otherwise specified. Interviewers told respondents to consider foods prepared at any home to be prepared at home and foods prepared at a restaurant or commercial food service establishment to be prepared outside the home. ‡All cases included were in nontravelers >1 y old; each serogroup-specific analysis had 2,365 noninfant, nontraveler controls. The overall number of cases for each serogroup-specific analysis is listed in the respective section header. During nearest-neighbors matching, cases and controls without a match were excluded for the exposure under consideration. Thus, the numbers of cases and controls that were matched and included in the analysis of each exposure is smaller than the total. §p adjusted for multiple testing using Benjamini-Hochberg-Yekutieli method.

## Discussion 

We found non-O157 STEC infections were associated with international travel and domestic exposure to a wide variety of foods and animal environments. Among 18 food consumption risks with site of consumption indicated, 94% were in restaurants. The wide variety of foods implicated suggests that sources of infection, and thus control measures, for non-157 STEC are more similar to those for *Salmonella* than to those for STEC O157 (*17*). Control measures focused on improving the food safety system, in particular for produce and restaurants, are likely to decrease illness the most. 

Our finding of large population-level risks attributable to eating at restaurants is notable because most food is consumed at home (*18*). FoodNet studies also identified restaurants as risks for STEC O157 (*19*) and *Campylobacter* (*20*) infections. A study from Australia linked non-O157 STEC illnesses to catered meals (*21*). In a review of US restaurant outbreaks, food handling and preparation practices were implicated in about half and food contaminated before entering the restaurant in about one quarter of *Salmonella* outbreaks (data for STEC not provided) (*22*,*23*). Policies that help promote a culture of food safety for restaurants include staff training in and oversight of food preparation and purchase agreement requirements that foods meet or exceed standards promoted by the Food Safety Modernization Act and the US Department of Agriculture’s Food Safety and Inspection Service. Health officials can also drive improved adherence to the Food and Drug Administration Food Code or stricter local regulations.

Our analysis indicated that eating lettuce, tomatoes, and other produce commonly consumed raw accounts for a large proportion of illnesses. One review of STEC found that row crop vegetables were associated with more outbreaks than any other food and significantly more non-O157 outbreaks than beef (*12*). Produce also transmits a high proportion of foodborne illnesses caused by other pathogens (*17*,*23*–*25*). Identifying particular growing areas and farms as sources of produce associated with outbreaks would provide a more efficient targeted process for preventing contamination before produce arrives at restaurants or stores (*26*). Produce growers, suppliers, sellers, and commercial establishments should adhere to guidelines to assure that produce is safe when purchased. The Food and Drug Administration is charged with implementing the Produce Safety Rule, part of the Food Safety Modernization Act, which includes requiring routine inspections of large produce farms. Best practice standards for biosecurity and water management should recognize the risk from environmental contamination caused by wildlife and from the use of untreated water contaminated with fecal matter from food-producing animals on crops (*26*,*27*). Preventing cross-contamination of produce from meat in restaurants and homes is also essential. 

Further regulatory measures could decrease transmission of non-O157 STEC. In 2012, similar to the practice for STEC O157 since 1994, the Food Safety and Inspection Service named the 6 non-O157 STEC serogroups (O26, O103, O111, O121, O145, and O45) most frequently linked to human illness as adulterants in raw, nonintact beef products (*28*). Although we observed inverse associations for some beef exposures, the consumption of any beef at a table service restaurant had a PAF of 10.1% and pink ground beef hamburger had an OR of 9, indicating those are high-risk exposures. We found eating ground beef hamburgers from fast-food restaurants was not associated with illness, similar to the finding of a FoodNet study of STEC O157 infections conducted during 1996–1997 (*19*). Those findings suggest that standard hamburger cooking procedures in fast-food restaurants are effective. PAFs of 16% for chicken and 10% for pork prepared in a restaurant suggest that those meats might transmit non-O157 STEC. US outbreaks caused by O157 but not non-O157 STEC have been linked to those foods (*29*).

We identified a wide variety of risky exposures related to infection from animals; visiting, living on, or working on a farm, petting zoo, or fair had the highest PAF (14.7%). Visiting (PAF 8.2%) and living on (PAF 5.2%) a farm each conferred risk. The study implicated specific animal types, including calves, chickens, cows, goats, horses, pigs, or sheep, as well as contact with horse feed and with wild deer or elk or their droppings. Contact with farm animals, particularly but not exclusively ruminants, or their environments is a known risk factor for both non-O157 (*20*,*21*,*27*,*30*) and O157 STEC infections (19,32,33). Handwashing is essential for preventing infections in these settings. Guidelines have been published for behaviors in public settings with animals (*34*); development of guidelines for nonpublic settings could help avert infections.

Although risk factors that have high PAFs provide the largest opportunities for reducing illnesses, many exposures had significantly high ORs, particularly animal contact and environmental exposures, which also signal potential targets for reducing infections. Very high ORs (6.8–66.9) indicating high individual-level risk were identified for exposure to environments with calves, cows, chickens, goats, horses, pigs, and sheep. Other exposures with high ORs (4.3–7.7) were, in descending order, eating raspberries in a restaurant, drinking untreated water, and eating cantaloupe in a restaurant. Drinking untreated water was also identified as a risk factor for O157 STEC infection in another FoodNet case–control study (*22*).

The similarity of serotypes in our study to those more recently causing illness indicates that the most notable risk factors we found likely remain current. The top 4 serogroups in our study, which accounted for 70% of isolates, were the same as the top 4 named adulterants in 2012. They were also the top 4 non-O157 STEC isolates reported to national surveillance during the study period (74% of isolates) and in the years with the most recently validated data, 2016–2018 (78% of isolates) (S. Browning, Centers for Disease Control and Prevention, December 18, 2020 email). The next 5 most common serogroups in our study were all among the top 11 serogroups nationally during the study period and 2016–2018. Regional variations in sources may influence serotype frequency but variations in laboratory practices may also affect frequency data (*35*,*36*). For example, some public health laboratories attempt to identify only the 3 most common serogroups, others test for the top 6, and others routinely send all isolates to CDC for serogrouping. It is possible that our study protocol requiring that all non-O157 STEC isolates be sent to CDC for serotyping resulted in recognition of illnesses caused by less common serogroups. 

Nearest-neighbor matching approaches have a solid theoretical basis in epidemiologic research (*37*–*39*), but applying this method to matching in case–control studies of enteric diseases is recent (*13*). Although it is impossible to account for every possible confounder when selecting controls, this approach allows the most closely matched controls to be selected for each case. The nearest-neighbor approach permitted better control of confounding and would be expected to produce less-biased estimates than our original scheme that matched only on age, sex, and geography. One apparent benefit of our study approach was that we did not observe the large number of spurious inverse effects for vegetable and fruit items that have been seen in other studies (*20*,*31*,*41*). 

Our study was limited to cases reported to public health departments and thus dependent on infected persons seeking health care and providers obtaining fecal specimens, so data may not be representative of all non-O157 STEC illnesses (*40*). We only enrolled patients residing in the FoodNet catchment area, which is not completely representative of the US population (*41*). In addition, patients were significantly more likely than controls to be Hispanic, possibly because controls were selected from purchased commercial lists of telephone numbers that included only landlines; persons of Hispanic ethnicity were more likely than others to live in households with only cellular telephones during the study (*42*). As in any case–control study, there were probably nondifferential information biases (e.g., differences in the way patients remember and report exposures compared with controls). Finally, unlike in outbreak investigations, in which a particular exposure can be confirmed as the source, associations in studies of sporadic infections do not confirm a particular source because of the possibility of residual confounding. Although we used an advanced method to control for confounding, residual confounding for some associations and for common coexposures was still likely. For example, many salads include both lettuce (PAF = 39.3%) and tomato (PAF = 21.3%); eating a tomato might be associated with illness only because it is consumed with contaminated lettuce. However, a major strength of studies of sporadic cases is that, unlike outbreak investigations, they can identify the exposures associated with the most illnesses in a population; conclusions about associations can be bolstered by information from outbreaks and microbiologic studies of sources. Studies such as ours can be used to target interventions that reduce the most illnesses in a population and evaluate the effectiveness of the intervention. 

In conclusion, sporadic non-O157 STEC infections were associated with a wide variety of food and farm animal environment-associated exposures, reflecting widespread carriage by animals. As for *Salmonella*, non-O157 STEC are a diverse group of organisms, widely distributed in food-producing and wild animals; many foods contaminated with animal feces transmit these pathogens. Therefore, non-O157 STEC infections might best be prevented by widespread improvements in food safety systems. To have the greatest effect in reducing the incidence of these infections, control measures should focus on decreasing contamination of produce consumed raw, especially lettuce, as well as improving the safety of food consumed in restaurants and decreasing transmission from animal environments. Such measures would also decrease illnesses caused by other enteric pathogens (*30*,*32*). Control measures that could be effective include decreasing carriage of pathogens by food animals, decreasing contamination of farm environments with food animal fecal matter, and decreasing contamination of foods of animal origin at slaughter. Transmission directly from farm animal environments could be decreased by improving hand hygiene; for example, by designing systems in which handwashing is the default behavior after exposure to those environments. 

Appendix Additional information about risk factors for non-O157 Shiga toxin–producing *Escherichia coli* infections, United States.
