# Supplementary material for: Risk Factors for Non-O157 Shiga Toxin–Producing Escherichia coli Infections, United States
Source: Emerg Infect Dis. 2023 Jun;29(6):1183–90. doi: 10.3201/eid2906.221521 (PMC10202860; doi:10.3201/eid2906.221521)
Supplement: Appendix — Additional information about risk factors for non-O157 Shiga toxin–producing Escherichia coli infections, United States. [file 22-1521-Techapp-s1.pdf]

Article DOI: https://doi.org/10.3201/eid2906.221521

EID cannot ensure accessibility for supplementary materials supplied by authors. Readers who have difficulty accessing supplementary content should contact the authors for assistance.

Risk Factors for Non-O157 Shiga Toxin–Producing *Escherichia coli* Infections, United States

Appendix

Appendix Table 1. Factors examined in case-control study of domestically acquired non-O157 Shiga toxin-producing *E. coli* infections among infants, with odds ratios (ORs)—FoodNet, 2012-2015

	Cases[†] (n=27)		Controls[†] (n=68)		Multivariable analysis		
Characteristic, food consumed, or exposure[*]	n/N[†]	(%)	n/N[†]	(%)	OR	(95% CI)	P[‡]
Demographic							
Asian	1/12	(8)	0/39	(0)	9.0	(0.1-3674.7)	1.00
Black	1/11	(9)	4/39	(10)	0.7	(0-6.2)	1.00
Hispanic	1/9	(11)	3/41	(7)	1.7	(0.1-17.6)	1.00
Multiracial	0/10	(0)	4/38	(11)	0.2	(0-3)	1.00
White	10/11	(91)	31/39	(79)	2.3	(0.4-26.9)	1.00
Dairy							
Ice cream	3/11	(27)	6/41	(15)	1.3	(0.2-7.5)	1.00
Prepared inside the home	3/11	(27)	5/42	(12)	1.8	(0.3-9.5)	1.00
Prepared outside the home	0/11	(0)	1/41	(2)	1.4	(0-49.9)	1.00
Pasteurized milk	1/11	(9)	4/42	(10)	0.7	(0-5.9)	1.00
Prepared inside the home	1/10	(10)	4/42	(10)	1.2	(0.1-11.6)	1.00
Soft cheese (e.g., feta, brie) prepared inside the home	0/11	(0)	1/41	(2)	0.3	(0-14.1)	1.00
Yogurt	1/7	(14)	5/40	(12)	1.5	(0.1-13.3)	1.00
Prepared inside the home	1/10	(10)	6/41	(15)	0.6	(0-4.5)	1.00
Fruit and Vegetable							
Apples	0/10	(0)	2/41	(5)	1.0	(0-26.8)	1.00
Prepared inside the home	0/11	(0)	2/41	(5)	0.9	(0-23.5)	1.00
Bananas	3/9	(33)	8/40	(20)	4.7	(0.6-52.0)	1.00
Prepared inside the home	4/14	(29)	8/40	(20)	0.9	(0.1-7.6)	1.00
Prepared outside the home	1/10	(10)	0/40	(0)	81.0	(2.0-29190.1)	1.00
Blueberries	1/11	(9)	0/41	(0)	57.0	(1.4-20687.9)	1.00
Prepared inside the home	1/12	(8)	0/41	(0)	15.0	(0.3-5804.6)	1.00
Prepared outside the home	1/12	(8)	0/41	(0)	15.0	(0.3-5804.6)	1.00
Carrots	2/11	(18)	0/41	(0)	42.9	(1.9-11827.7)	1.00
Prepared inside the home	2/12	(17)	0/41	(0)	18.0	(0.8-2072.8)	1.00
Exotic fruit (e.g., kiwi, avocado)	1/8	(12)	3/41	(7)	3.8	(0.3-65.5)	1.00
Prepared inside the home	1/12	(8)	3/40	(8)	0.2	(0-5.6)	1.00
Grapes	1/8	(12)	1/41	(2)	17.0	(0.6-3076.9)	1.00
Prepared inside the home	1/12	(8)	1/42	(2)	2.1	(0.1-41.2)	1.00
Lettuce	1/11	(9)	0/41	(0)	39.0	(0.9-14310.8)	1.00
Oranges	1/9	(11)	1/41	(2)	3.4	(0.1-70.0)	1.00
Prepared inside the home	1/12	(8)	1/42	(2)	1.9	(0.1-38.5)	1.00
Organic produce	1/8	(12)	3/39	(8)	3.0	(0.2-42.3)	1.00
Other fruit (not citrus, pears, apples, tree fruit, strawberries, raspberries, grapes, bananas, cantaloupe, watermelon, honeydew, pineapple, or exotic fruit)	1/11	(9)	0/41	(0)	9.0	(0.1-3674.7)	1.00
Prepared outside the home	1/12	(8)	0/41	(0)	9.0	(0.1-3674.7)	1.00
Pears	0/10	(0)	1/41	(2)	1.9	(0-64.1)	1.00
Prepared inside the home	1/11	(9)	1/41	(2)	3.4	(0.1-70.0)	1.00
Raspberries	1/11	(9)	0/41	(0)	39.0	(0.9-14310.8)	1.00
Strawberries	0/10	(0)	1/41	(2)	1.9	(0-64.1)	1.00
Prepared inside the home	0/11	(0)	1/41	(2)	1.7	(0-57.0)	1.00
Tree fruit other than citrus, pears, or apples (e.g., apricot, nectarine, peach, plum)	1/9	(11)	2/41	(5)	2.2	(0.1-36.3)	1.00
Prepared inside the home	1/12	(8)	2/42	(5)	1.0	(0.1-18.1)	1.00
Watermelon	0/12	(0)	1/42	(2)	0.1	(0-6.8)	1.00
Prepared inside the home	0/12	(0)	2/43	(5)	0.4	(0-9.0)	1.00
Meat, Poultry, Pork, and Seafood							
Beef	1/10	(10)	1/40	(2)	6.3	(0.3-119.9)	1.00
Prepared at home	0/9	(0)	1/40	(2)	2.3	(0-78.4)	1.00
Chicken	0/9	(0)	5/41	(12)	0.1	(0-1.6)	1.00
Prepared inside the home	0/9	(0)	5/41	(12)	0.2	(0-3.6)	1.00
Ground beef at a fast-food restaurant	1/10	(10)	0/40	(0)	45.0	(1.1-16436.6)	1.00
Ground beef hamburger prepared at a fast-food restaurant	1/10	(10)	0/40	(0)	57.0	(1.4-20687.9)	1.00
Ground beef other than hamburgers	0/10	(0)	2/41	(5)	0.6	(0-11.3)	1.00
Prepared at a fast-food restaurant	3/12	(25)	0/40	(0)	37.1	(2.4-1646.2)	1.00
Handled meat (e.g., beef, pork, poultry) or fish in past 3 months	3/8	(38)	6/40	(15)	3.7	(0.6-25.7)	1.00
Household member handled raw beef in past 3 months	1/13	(8)	4/40	(10)	0.2	(0-5.3)	1.00
Organic meat	1/12	(8)	1/38	(3)	1.9	(0.1-38.5)	1.00
Other meat, poultry, or fish (not chicken, turkey, pork, lamb, veal, jerky, venison, elk, goat, bison, salami, pepperoni, summer sausage, shrimp, shellfish, or raw fish/sushi) prepared inside the home	0/10	(0)	1/41	(2)	2.3	(0-78.4)	1.00
Pork prepared inside the home	0/10	(0)	1/41	(2)	2.3	(0-78.4)	1.00
Salami prepared inside the home	1/10	(10)	0/40	(0)	9.0	(0.1-3674.7)	1.00
Turkey prepared inside the home	0/10	(0)	2/42	(5)	0.4	(0-10.4)	1.00
Venison	0/9	(0)	1/40	(2)	1.4	(0-49.9)	1.00
Prepared inside the home	0/10	(0)	1/40	(2)	1.2	(0-42.8)	1.00
Environmental							
Camping	1/11	(9)	1/41	(2)	7.2	(0.4-130.6)	1.00
Contact with animal-based dog treats (e.g., rawhides, pig's ear)	0/8	(0)	1/39	(3)	1.7	(0-57.0)	1.00
Contact with animals	2/10	(20)	25/41	(61)	0.1	(0-0.7)	1.00
Contact with any wild animals or their droppings	1/11	(9)	0/41	(0)	15.0	(0.3-5804.6)	1.00
Contact with birds	0/9	(0)	1/41	(2)	1.0	(0-35.6)	1.00
Contact with cats	1/10	(10)	13/41	(32)	0.4	(0-2.3)	1.00
Contact with chickens	0/9	(0)	1/41	(2)	1.9	(0-64.1)	1.00
Contact with dog food	1/11	(9)	0/42	(0)	9.0	(0.1-3674.7)	1.00
Contact with dogs	2/11	(18)	22/43	(51)	0.3	(0.1-1.3)	1.00
Household member visited/worked on farm with animals	1/12	(8)	3/40	(8)	1.2	(0-554.8)	1.00
Household member visited/worked on farm with cows	1/13	(8)	3/40	(8)	1.0	(0-8290.8)	1.00
Live on a farm	2/14	(14)	4/41	(10)	2.6	(0.1-390.7)	1.00
With calves present	1/13	(8)	1/40	(2)	3.0	(0.1-612.5)	1.00
With chickens present	0/13	(0)	2/41	(5)	0.3	(0-10.1)	1.00
With cows present	2/12	(17)	3/40	(8)	3.6	(0.3-36.6)	1.00
Live on, visit, or work on a farm, petting zoo, or fair	3/12	(25)	5/40	(12)	51.0	(1.2-18562.2)	1.00
And have contact with chickens	0/12	(0)	1/40	(2)	1.0	(0-35.6)	1.00
With calves present	2/11	(18)	1/39	(3)	69.0	(1.7-24939.1)	1.00
With chickens present	0/13	(0)	2/40	(5)	0.2	(0-6.7)	1.00
With cows or calves present	2/12	(17)	5/40	(12)	1.5	(0.1-31.6)	1.00
With cows present	2/12	(17)	5/40	(12)	1.3	(0.1-10.4)	1.00
With turkeys present	0/13	(0)	1/40	(2)	0.6	(0-21.3)	1.00
Visit a fair	0/14	(0)	1/41	(2)	0.6	(0-21.3)	1.00
Visit a farm with cows present	0/13	(0)	1/42	(2)	1.7	(0-57.0)	1.00
Visit a petting zoo	1/12	(8)	0/40	(0)	33.0	(0.8-12184.8)	1.00

Characteristic, food consumed, or exposure*	Cases† (n=27) n/N†	(%)	Controls† (n=68) n/N†	(%)	Multivariable analysis OR	(95% CI)	P ‡
Work, play, or help in garden	1/12	(8)	0/41	(0)	39.0	(0.9-14310.8)	1.00
Water							
Drink bottled water	8/12	(67)	22/38	(58)	1.2	(0.3-5.0)	1.00
Drink untreated water (e.g., lake, spring, or river)	0/10	(0)	1/39	(3)	1.4	(0-49.9)	1.00
Swim or play	NA	NA	NA	NA	NA	NA	NA
In pool	0/11	(0)	2/41	(5)	1.1	(0-18.8)	1.00
In water	0/11	(0)	3/41	(7)	0.2	(0-4.5)	1.00
In water park	0/12	(0)	1/41	(2)	1.4	(0-49.9)	1.00
Treatment for well water at home other than with a whole-house point-of-entry device	0/13	(0)	2/41	(5)	0.8	(0-14.8)	1.00
Use municipal water at home	3/9	(33)	14/40	(35)	1.5	(0.2-11.3)	1.00
Use municipal water away from home	1/8	(12)	6/33	(18)	0.7	(0.1-5.3)	1.00
Use private well water at home	3/13	(23)	5/41	(12)	2.9	(0.3-26.1)	1.00
Use private well water away from home	1/10	(10)	0/34	(0)	15.0	(0.3-5804.6)	1.00
Whole house point-of-entry treatment for well water at home	0/12	(0)	2/40	(5)	0.7	(0-12.8)	1.00
Other							
Antibiotics prior to illness	2/11	(18)	2/40	(5)	10.1	(1.0-144.1)	1.00
Attend childcare setting or center	1/10	(10)	9/41	(22)	0.6	(0.1-3.5)	1.00
Attend, work, or volunteer at a childcare center	5/11	(45)	5/40	(12)	4.5	(1.0-22.1)	1.00
Children under 5 years of age in household	3/12	(25)	17/40	(42)	0.5	(0.1-1.8)	1.00
Contact with someone with diarrheal illness	1/9	(11)	1/38	(3)	4.1	(0.2-79.4)	1.00
Eat at a fast-food restaurant	2/13	(15)	0/40	(0)	27.7	(1.3-4327.9)	1.00
Eat at a table service restaurant	1/11	(9)	0/40	(0)	15.0	(0.3-5804.6)	1.00
Live, work, or visit residential facility (e.g., nursing home, hospital)	1/9	(11)	3/40	(8)	3.0	(0.2-44.4)	1.00
Stomach acid-reducing medications in 4 weeks before illness	1/11	(9)	3/41	(7)	1.3	(0.1-10.8)	1.00
Travel in state of residence	1/10	(10)	8/39	(21)	0.6	(0-3.8)	1.00
Travel outside state of residence	1/13	(8)	4/42	(10)	1.2	(0.1-9.7)	1.00

*In the 7 days before illness began unless otherwise specified. Only exposures with sufficient sample sizes for analysis after nearest-neighbors matching are listed. Interviewers told respondents to consider foods prepared at any home to be prepared at home and foods prepared at a restaurant or commercial food service establishment to be prepared outside the home.
†The initial sample for each exposure was 27 cases and 68 controls. During nearest-neighbors matching, cases and controls without a match were excluded for the exposure under consideration. Thus, the numbers (i.e., N in the table) of cases and controls that were matched and included in the analysis of each exposure is smaller than the total. Number of exposed is denoted by n. Nineteen controls did not match to any case for any exposure. They were different from cases: most reported ≥30 exposures whereas most cases reported <25 exposures.
‡P is adjusted for multiple testing using the Benjamini-Hochberg-Yekutieli method

Appendix Table 2. All factors examined in case-control study of domestically acquired non-O157 Shiga toxin-producing *E. coli* infections, with odds ratios (ORs)— FoodNet, 2012-2015

Characteristic or exposure*	Cases† (n=774) n/N†	(%)	Controls† (n=2,365) n/N†	(%)	Multivariable analysis OR	(95% CI)	P ‡
Demographic							
American Indian or Alaskan Native	2/404	(0)	10/1204	(1)	0.8	(0.2-3.3)	1.00
Asian	9/407	(2)	25/1198	(2)	1.1	(0.4-2.7)	1.00
Black	19/410	(5)	94/1205	(8)	0.5	(0.3-0.8)	0.19
Hispanic	76/425	(18)	114/1256	(9)	2.9	(2.0-4.3)	<0.001
Multiracial	12/413	(3)	29/1201	(2)	1.0	(0.5-2.1)	1.00
White	369/411	(90)	1049/1207	(87)	1.5	(1.0-2.4)	0.7
Dairy							
Cheese curds	16/434	(4)	33/1272	(3)	1.4	(0.7-2.7)	1.00
Prepared inside the home	8/439	(2)	28/1279	(2)	0.7	(0.3-1.7)	1.00
Cheese other than hard cheese, soft cheese, Mexican-style cheese, or cheese curds	117/444	(26)	308/1287	(24)	1.2	(0.9-1.6)	1.00
Prepared inside the home	84/437	(19)	267/1284	(21)	0.9	(0.7-1.3)	1.00
Hard cheese (e.g., gouda, cheddar)	271/443	(61)	824/1274	(65)	0.9	(0.7-1.2)	1.00
Prepared inside the home	173/449	(39)	637/1267	(50)	0.6	(0.5-0.8)	0.007
Ice cream	202/441	(46)	686/1276	(54)	0.7	(0.5-0.9)	0.07
Prepared inside the home	165/451	(37)	639/1281	(50)	0.6	(0.5-0.8)	0.003
Prepared outside the home	81/440	(18)	219/1274	(17)	1.0	(0.7-1.5)	1.00
Pasteurized milk	325/450	(72)	1046/1284	(81)	0.5	(0.4-0.7)	<0.001
Prepared inside the home	308/449	(69)	1025/1287	(80)	0.5	(0.4-0.7)	<0.001
Prepared outside the home	67/448	(15)	248/1282	(19)	0.6	(0.4-0.9)	0.12
Queso fresco or Mexican-style cheese	49/439	(11)	119/1281	(9)	1.4	(0.9-2.1)	1.00
Prepared inside the home	32/439	(7)	80/1288	(6)	1.3	(0.8-2.1)	1.00
Raw milk	7/431	(2)	5/1270	(0)	3.4	(1.0-12.7)	0.93
Prepared inside the home	7/435	(2)	6/1271	(0)	2.6	(0.8-9.1)	1.00
Prepared outside the home	1/430	(0)	0/1270	(0)	27.0	(0.6-10058.7)	1.00
Soft cheese (e.g., feta, brie)	115/440	(26)	350/1275	(27)	0.9	(0.7-1.2)	1.00
Prepared inside the home	80/440	(18)	285/1290	(22)	0.8	(0.6-1.1)	1.00
Unpasteurized cheese	5/435	(1)	9/1272	(1)	1.3	(0.4-4.7)	1.00
Prepared inside the home	6/437	(1)	5/1273	(0)	3.5	(0.9-15.1)	1.00
Prepared outside the home	0/434	(0)	2/1272	(0)	0.1	(0-7.4)	1.00
Yogurt	239/437	(55)	748/1279	(58)	1.0	(0.8-1.3)	1.00
Prepared inside the home	232/462	(50)	737/1286	(57)	0.8	(0.6-1.1)	1.00
Prepared outside the home	41/437	(9)	84/1289	(7)	1.7	(1.1-2.7)	0.42
Fruit and Vegetable							
Alfalfa sprouts	7/433	(2)	6/1273	(0)	3.9	(1.1-14.2)	0.65
Prepared inside the home	6/432	(1)	5/1274	(0)	2.9	(0.8-11.2)	1.00
Prepared outside the home	3/434	(1)	2/1275	(0)	3.5	(0.5-25.6)	1.00
Apples	276/443	(62)	903/1276	(71)	0.8	(0.6-1.1)	1.00
Prepared inside the home	246/431	(57)	871/1261	(69)	0.6	(0.4-0.8)	0.007
Prepared outside the home	54/434	(12)	117/1263	(9)	1.4	(0.9-2.2)	1.00
Bananas	316/443	(71)	953/1287	(74)	1.1	(0.8-1.4)	1.00
Prepared inside the home	303/447	(68)	916/1272	(72)	1.0	(0.8-1.4)	1.00
Prepared outside the home	37/434	(9)	68/1267	(5)	1.8	(1.1-3.1)	0.47
Bean sprouts	12/432	(3)	12/1271	(1)	4.2	(1.6-11.1)	0.11
Prepared inside the home	8/432	(2)	9/1272	(1)	4.1	(1.3-12.6)	0.35
Blueberries	128/445	(29)	377/1280	(29)	1.2	(0.9-1.6)	1.00
Prepared inside the home	114/455	(25)	370/1283	(29)	1.0	(0.7-1.3)	1.00
Prepared outside the home	11/451	(2)	23/1276	(2)	1.5	(0.6-3.5)	1.00
Broccoli	103/434	(24)	360/1279	(28)	1.0	(0.7-1.4)	1.00
Prepared inside the home	100/443	(23)	339/1281	(26)	1.0	(0.7-1.3)	1.00
Prepared outside the home	14/429	(3)	54/1279	(4)	0.7	(0.3-1.4)	1.00
Cabbage	56/445	(13)	145/1280	(11)	1.1	(0.8-1.7)	1.00
Prepared inside the home	38/454	(8)	113/1284	(9)	1.1	(0.7-1.8)	1.00
Prepared outside the home	23/442	(5)	53/1284	(4)	1.3	(0.7-2.2)	1.00
Cantaloupe	100/436	(23)	258/1272	(20)	1.6	(1.1-2.2)	0.15
Prepared inside the home	88/442	(20)	257/1281	(20)	1.2	(0.9-1.7)	1.00
Prepared outside the home	18/431	(4)	22/1274	(2)	4.3	(1.9-9.9)	0.02
Carrots	217/456	(48)	699/1281	(55)	0.8	(0.6-1.1)	1.00
Prepared inside the home	182/438	(42)	654/1270	(51)	0.7	(0.5-0.9)	0.2
Prepared outside the home	50/456	(11)	93/1278	(7)	1.7	(1.1-2.6)	0.3
Celery	93/441	(21)	287/1267	(23)	1.1	(0.8-1.6)	1.00
Prepared inside the home	84/437	(19)	272/1267	(21)	1.2	(0.8-1.6)	1.00
Prepared outside the home	20/436	(5)	30/1271	(2)	2.5	(1.3-4.8)	0.22
Cilantro	67/443	(15)	149/1281	(12)	1.8	(1.2-2.6)	0.1
Prepared inside the home	48/448	(11)	137/1285	(11)	1.2	(0.8-1.9)	1.00
Prepared outside the home	17/444	(4)	23/1282	(2)	3.0	(1.4-6.2)	0.12
Citrus other than oranges	99/436	(23)	302/1279	(24)	1.1	(0.8-1.5)	1.00
Prepared inside the home	89/462	(19)	287/1282	(22)	0.9	(0.7-1.2)	1.00
Prepared outside the home	20/434	(5)	38/1275	(3)	1.6	(0.8-3.0)	1.00
Cucumbers	158/449	(35)	458/1266	(36)	1.2	(0.9-1.7)	1.00
Prepared inside the home	125/435	(29)	418/1261	(33)	1.0	(0.8-1.4)	1.00
Prepared outside the home	43/441	(10)	85/1264	(7)	1.8	(1.1-2.9)	0.38
Exotic fruit (e.g., kiwi, avocado)	140/444	(32)	336/1274	(26)	1.7	(1.3-2.3)	0.02
Prepared inside the home	124/448	(28)	322/1267	(25)	1.5	(1.1-2.0)	0.14
Prepared outside the home	35/449	(8)	30/1275	(2)	3.9	(2.1-7.1)	<0.001
Fresh herbs other than parsley or cilantro	35/437	(8)	95/1271	(7)	1.5	(0.9-2.5)	1.00

Characteristic or exposure*	Cases† (n=774) n/N†	(%)	Controls† (n=2,365) n/N†	(%)	Multivariable analysis OR	(95% CI)	P ‡
Prepared inside the home	29/436	(7)	93/1274	(7)	1.3	(0.8-2.1)	1.00
Prepared outside the home	5/434	(1)	3/1270	(0)	6.3	(1.3-31.5)	0.43
Grapes	223/445	(50)	701/1276	(55)	1.0	(0.8-1.3)	1.00
Prepared inside the home	197/451	(44)	680/1279	(53)	0.8	(0.6-1.0)	0.81
Prepared outside the home	36/443	(8)	70/1266	(6)	1.8	(1.1-3.1)	0.51
Green onions	62/442	(14)	163/1276	(13)	1.5	(1.0-2.2)	1.00
Prepared inside the home	57/453	(13)	152/1272	(12)	1.3	(0.9-1.9)	1.00
Prepared outside the home	22/443	(5)	25/1275	(2)	2.8	(1.4-5.6)	0.09
Homegrown vegetables	73/442	(17)	165/1285	(13)	1.5	(1.0-2.1)	0.51
Honeydew	30/439	(7)	78/1276	(6)	1.5	(0.9-2.6)	1.00
Prepared inside the home	23/440	(5)	74/1278	(6)	1.2	(0.7-2.0)	1.00
Prepared outside the home	11/437	(3)	10/1277	(1)	5.2	(1.8-14.9)	0.07
Iceberg lettuce	159/426	(37)	406/1238	(33)	1.6	(1.2-2.2)	0.12
Prepared inside the home	118/425	(28)	355/1239	(29)	1.1	(0.8-1.5)	1.00
Prepared outside the home	86/415	(21)	156/1245	(13)	2.7	(1.8-3.9)	<0.001
Lettuce	288/447	(64)	710/1268	(56)	2.6	(1.8-3.6)	<0.001
Lettuce other than iceberg or romaine	-	-	-	-	-	-	-
Prepared inside the home	34/418	(8)	77/1230	(6)	1.8	(1.0-2.9)	0.6
Prepared outside the home	9/425	(2)	17/1230	(1)	1.7	(0.7-4.4)	1.00
Mixed greens (e.g., spring mix, swiss chard)	69/429	(16)	208/1282	(16)	1.4	(0.9-2.0)	1.00
Prepared inside the home	57/436	(13)	187/1285	(15)	1.1	(0.7-1.7)	1.00
Prepared outside the home	16/428	(4)	41/1282	(3)	1.3	(0.6-2.6)	1.00
Onions other than white or red	161/453	(36)	438/1267	(35)	1.5	(1.1-2.0)	0.21
Prepared inside the home	123/438	(28)	403/1260	(32)	1.0	(0.8-1.4)	1.00
Prepared outside the home	54/438	(12)	87/1260	(7)	2.5	(1.6-4.0)	0.006
Oranges	144/438	(33)	476/1272	(37)	1.0	(0.7-1.3)	1.00
Prepared inside the home	124/444	(28)	459/1272	(36)	0.8	(0.6-1.1)	1.00
Prepared outside the home	19/436	(4)	53/1273	(4)	1.2	(0.6-2.2)	1.00
Organic produce	105/439	(24)	284/1280	(22)	1.5	(1.1-2.0)	0.23
Other fruit (not citrus, pears, apples, tree fruit, strawberries, raspberries, grapes, bananas, cantaloupe, watermelon, honeydew, pineapple, or exotic fruit)	29/445	(7)	52/1280	(4)	1.9	(1.1-3.2)	0.46
Prepared inside the home	25/448	(6)	54/1278	(4)	1.6	(0.9-2.7)	1.00
Prepared outside the home	4/441	(1)	3/1279	(0)	4.4	(0.8-23.8)	1.00
Parsley	37/441	(8)	79/1275	(6)	2.3	(1.3-3.8)	0.09
Prepared inside the home	34/438	(8)	77/1278	(6)	2.2	(1.3-3.8)	0.1
Prepared outside the home	4/445	(1)	8/1268	(1)	1.5	(0.4-5.3)	1.00
Pea pods	25/443	(6)	106/1273	(8)	0.6	(0.4-1.1)	1.00
Prepared inside the home	24/443	(5)	108/1280	(8)	0.7	(0.4-1.1)	1.00
Prepared outside the home	4/445	(1)	9/1273	(1)	0.9	(0.2-3.4)	1.00
Pears	54/439	(12)	241/1268	(19)	0.6	(0.4-0.9)	0.17
Prepared inside the home	50/443	(11)	246/1278	(19)	0.5	(0.4-0.8)	0.02
Prepared outside the home	6/436	(1)	18/1268	(1)	1.3	(0.4-3.9)	1.00
Peppers	117/439	(27)	370/1277	(29)	1.3	(1.0-1.8)	1.00
Prepared inside the home	102/450	(23)	352/1276	(28)	0.9	(0.7-1.3)	1.00
Prepared outside the home	41/440	(9)	56/1274	(4)	3.0	(1.8-5.0)	0.002
Pineapple	98/449	(22)	221/1279	(17)	1.8	(1.3-2.6)	0.02
Prepared inside the home	80/446	(18)	206/1275	(16)	1.4	(1.0-2.0)	0.84
Prepared outside the home	23/440	(5)	29/1274	(2)	3.6	(1.8-7.1)	0.02
Prepackaged iceberg lettuce	73/371	(20)	212/1145	(19)	1.3	(0.9-1.9)	1.00
Prepackaged lettuce other than iceberg or romaine	22/418	(5)	42/1220	(3)	1.9	(1.0-3.5)	0.7
Prepackaged mixed greens (e.g., spring mix, swiss chard)	49/424	(12)	157/1253	(13)	1.2	(0.8-1.9)	1.00
Prepackaged raw spinach	60/432	(14)	184/1232	(15)	1.1	(0.8-1.6)	1.00
Prepackaged romaine	76/379	(20)	220/1145	(19)	1.3	(0.9-1.9)	1.00
Radishes	23/437	(5)	59/1277	(5)	1.4	(0.7-2.5)	1.00
Prepared inside the home	20/440	(5)	58/1279	(5)	1.2	(0.6-2.2)	1.00
Prepared outside the home	6/438	(1)	8/1279	(1)	2.0	(0.6-6.8)	1.00
Raspberries	81/437	(19)	249/1271	(20)	1.3	(0.9-1.8)	1.00
Prepared inside the home	76/439	(17)	260/1284	(20)	1.1	(0.8-1.5)	1.00
Prepared outside the home	11/432	(3)	10/1270	(1)	7.7	(2.4-27.5)	0.03
Raw spinach	92/441	(21)	243/1279	(19)	1.8	(1.2-2.5)	0.08
Prepared inside the home	74/451	(16)	223/1280	(17)	1.3	(0.9-1.8)	1.00
Prepared outside the home	30/445	(7)	44/1286	(3)	2.9	(1.7-5.2)	0.02
Romaine lettuce	131/427	(31)	369/1228	(30)	1.4	(1.0-1.9)	1.00
Prepared inside the home	105/425	(25)	338/1219	(28)	1.1	(0.8-1.5)	1.00
Prepared outside the home	51/431	(12)	95/1227	(8)	2.2	(1.4-3.4)	0.03
Salsa	71/442	(16)	137/1275	(11)	1.9	(1.3-2.8)	0.04
Prepared inside the home	41/452	(9)	109/1276	(9)	1.3	(0.8-2.0)	1.00
Prepared outside the home	37/449	(8)	53/1277	(4)	2.1	(1.3-3.3)	0.12
Sprouts other than alfalfa or bean	3/429	(1)	6/1270	(0)	1.4	(0.3-5.9)	1.00
Prepared inside the home	3/428	(1)	6/1270	(0)	1.4	(0.3-5.9)	1.00
Strawberries	227/434	(52)	628/1269	(49)	1.5	(1.2-2.0)	0.1
Prepared inside the home	218/438	(50)	602/1265	(48)	1.4	(1.0-1.8)	0.47
Prepared outside the home	31/421	(7)	56/1265	(4)	2.2	(1.2-3.8)	0.17
Tomatoes	227/435	(52)	605/1265	(48)	1.7	(1.3-2.3)	0.03
Prepared inside the home	180/427	(42)	536/1253	(43)	1.3	(1.0-1.7)	1.00
Prepared outside the home	101/443	(23)	175/1266	(14)	2.5	(1.8-3.5)	<0.001
Tree fruit other than citrus, pears, or apples (e.g., apricot, nectarine, peach, plum)	101/447	(23)	274/1286	(21)	1.3	(1.0-1.8)	1.00
Prepared inside the home	88/447	(20)	268/1288	(21)	1.2	(0.8-1.6)	1.00
Prepared outside the home	9/447	(2)	22/1284	(2)	0.9	(0.4-2.1)	1.00
Unpasteurized apple cider or juice	8/434	(2)	8/1270	(1)	3.0	(1.0-9.1)	0.84
Unpasteurized juice other than apple cider or apple juice	7/431	(2)	8/1270	(1)	2.5	(0.8-8.2)	1.00
Watermelon	145/448	(32)	302/1276	(24)	2.4	(1.8-3.4)	<0.001
Prepared inside the home	120/445	(27)	291/1274	(23)	1.7	(1.2-2.3)	0.03
Prepared outside the home	22/446	(5)	34/1270	(3)	2.7	(1.3-5.5)	0.16
Other							
Antibiotics prior to illness	35/438	(8)	83/1265	(7)	1.3	(0.8-2.0)	1.00
Attend childcare setting or center	43/432	(10)	167/1251	(13)	0.7	(0.5-1.0)	1.00
Attend, work, or volunteer at a childcare center	63/438	(14)	198/1259	(16)	0.9	(0.6-1.2)	1.00
Children under 5 years of age in household	121/445	(27)	381/1253	(30)	0.8	(0.6-1.1)	1.00
Contact with someone with diarrheal illness	56/408	(14)	55/1153	(5)	3.6	(2.2-5.7)	<0.001
Eat at a fast-food restaurant	245/434	(56)	580/1278	(45)	1.7	(1.3-2.2)	0.02
Eat at a table service restaurant	207/454	(46)	425/1276	(33)	1.7	(1.3-2.3)	0.002
Live, work, or visit residential facility (e.g., nursing home, hospital)	55/446	(12)	100/1274	(8)	1.6	(1.1-2.4)	0.4
Stomach acid-reducing medications in 4 weeks before illness	93/430	(22)	174/1261	(14)	2.1	(1.5-2.9)	<0.001
Travel in state of residence	133/439	(30)	317/1261	(25)	1.4	(1.0-1.8)	0.47
Travel outside state of residence	63/447	(14)	113/1262	(9)	1.6	(1.1-2.3)	0.43
Meat, Poultry, Pork, and Seafood							
Beef	318/435	(73)	911/1264	(72)	1.0	(0.7-1.5)	1.00
Prepared at home	240/437	(55)	827/1257	(66)	0.6	(0.4-0.8)	0.004
Prepared at table service restaurant	85/443	(19)	135/1258	(11)	2.1	(1.5-3.0)	0.003
Bison	7/434	(2)	6/1265	(0)	5.5	(1.7-18.3)	0.15
Prepared inside the home	4/433	(1)	7/1266	(1)	2.2	(0.5-7.9)	1.00
Prepared outside the home	2/431	(0)	1/1263	(0)	7.4	(0.7-104.0)	1.00
Chicken	364/431	(84)	1045/1272	(82)	1.5	(1.0-2.2)	0.63
Prepared inside the home	303/445	(68)	955/1269	(75)	0.8	(0.6-1.0)	1.00
Prepared outside the home	203/446	(46)	465/1269	(37)	1.6	(1.2-2.0)	0.04
Elk	9/433	(2)	5/1268	(0)	6.2	(1.9-22.5)	0.09
Prepared inside the home	7/431	(2)	5/1266	(0)	4.1	(1.2-14.6)	0.48
Prepared outside the home	2/431	(0)	0/1267	(0)	18.0	(0.8-2072.8)	1.00
Goat	8/436	(2)	5/1267	(0)	5.3	(1.5-20.1)	0.2
Prepared inside the home	5/431	(1)	5/1266	(0)	3.4	(0.8-14.4)	1.00
Prepared outside the home	2/432	(0)	1/1266	(0)	6.9	(0.7-96.3)	1.00

Characteristic or exposure*	Cases† (n=774) n/N†	(%)	Controls† (n=2,365) n/N†	(%)	Multivariable analysis OR	(95% CI)	P ‡
Ground beef at a fast-food restaurant	120/420	(29)	309/1247	(25)	1.2	(0.9-1.6)	1.00
Ground beef hamburger	-	-	-	-	-	-	-
Prepared at a fast-food restaurant	104/431	(24)	287/1256	(23)	0.9	(0.7-1.3)	1.00
Prepared at home	141/445	(32)	550/1281	(43)	0.5	(0.4-0.7)	<0.001
Prepared at a table service restaurant	44/445	(10)	73/1276	(6)	2.4	(1.5-3.8)	0.01
Ground beef other than hamburgers	144/445	(32)	555/1282	(43)	0.6	(0.4-0.7)	0.002
Prepared at a fast-food restaurant	45/425	(11)	111/1255	(9)	1.3	(0.8-2.0)	1.00
Handled meat (e.g., beef, pork, poultry, or fish) in past 3 months	428/436	(98)	1216/1265	(96)	2.3	(1.0-5.7)	0.78
Handled raw steaks or intact cut of beef in past 3 months	54/437	(12)	208/1283	(16)	0.7	(0.5-1.0)	0.64
Household member handled raw beef in past 3 months	187/439	(43)	646/1276	(51)	0.7	(0.5-0.9)	0.09
Household member handled raw ground beef in past 3 months	87/457	(19)	318/1280	(25)	0.7	(0.5-1.0)	0.81
Intact cut of beef other than steak	49/456	(11)	203/1270	(16)	0.6	(0.4-0.8)	0.12
prepared at table service restaurant	14/453	(3)	18/1271	(1)	1.9	(0.9-4.2)	1.00
Jerky	47/435	(11)	73/1273	(6)	2.1	(1.4-3.4)	0.05
Prepared inside the home	28/432	(6)	48/1274	(4)	1.7	(1.0-2.9)	1.00
Prepared outside the home	18/441	(4)	37/1273	(3)	1.1	(0.6-2.1)	1.00
Lamb	11/435	(3)	21/1270	(2)	1.4	(0.6-3.2)	1.00
Prepared inside the home	4/433	(1)	12/1270	(1)	1.2	(0.3-4.1)	1.00
Prepared outside the home	8/428	(2)	11/1271	(1)	1.7	(0.6-4.8)	1.00
Organic meat	49/436	(11)	191/1280	(15)	0.8	(0.5-1.1)	1.00
Other meat, poultry, or fish (not chicken, turkey, pork, lamb, veal, jerky, venison, elk, goat, bison, salami, pepperoni, summer sausage, shrimp, shellfish, or raw fish/sushi)	82/449	(18)	189/1280	(15)	1.2	(0.9-1.7)	1.00
Prepared inside the home	57/447	(13)	180/1284	(14)	0.9	(0.6-1.2)	1.00
Prepared outside the home	22/444	(5)	33/1279	(3)	1.5	(0.8-2.7)	1.00
Pepperoni	149/443	(34)	425/1282	(33)	0.9	(0.7-1.2)	1.00
Prepared inside the home	63/442	(14)	254/1278	(20)	0.6	(0.4-0.9)	0.1
Prepared outside the home	99/444	(22)	229/1284	(18)	1.3	(0.9-1.8)	1.00
Pink ground beef hamburger	-	-	-	-	-	-	-
Prepared at a fast-food restaurant	7/425	(2)	11/1235	(1)	1.7	(0.6-5.0)	1.00
Prepared at a table service restaurant	17/443	(4)	7/1280	(1)	9.0	(3.5-24.7)	<0.001
Prepared inside the home	21/439	(5)	86/1265	(7)	0.7	(0.4-1.1)	1.00
Pink ground beef other than hamburger	12/436	(3)	41/1267	(3)	0.8	(0.4-1.5)	1.00
Prepared at a fast-food restaurant	2/418	(0)	1/1240	(0)	7.0	(0.7-97.5)	1.00
Pink intact cut of beef	4/455	(1)	26/1268	(2)	0.4	(0.1-1.2)	1.00
Pink pre-made frozen hamburger patties	4/443	(1)	23/1262	(2)	0.4	(0.1-1.2)	1.00
Pink steak	35/443	(8)	157/1274	(12)	0.6	(0.4-0.9)	0.37
Prepared at a table service restaurant	11/439	(3)	27/1278	(2)	1.5	(0.7-3.2)	1.00
Pork	189/447	(42)	471/1273	(37)	1.3	(1.0-1.7)	0.75
Prepared inside the home	139/447	(31)	434/1265	(34)	0.9	(0.7-1.1)	1.00
Prepared outside the home	71/450	(16)	86/1276	(7)	2.9	(1.9-4.2)	<0.001
Pre-made frozen hamburger patties	35/446	(8)	162/1269	(13)	0.6	(0.4-0.9)	0.17
Raw fish or sushi	25/440	(6)	25/1269	(2)	3.0	(1.5-5.9)	0.07
Prepared inside the home	2/433	(0)	6/1270	(0)	1.8	(0.3-8.7)	1.00
Prepared outside the home	24/435	(6)	22/1270	(2)	3.1	(1.6-6.3)	0.05
Salami	38/441	(9)	102/1265	(8)	1.0	(0.6-1.6)	1.00
Prepared inside the home	22/433	(5)	87/1269	(7)	0.8	(0.4-1.3)	1.00
Prepared outside the home	15/437	(3)	28/1263	(2)	1.7	(0.8-3.4)	1.00
Sausage other than summer sausage	85/440	(19)	240/1281	(19)	0.9	(0.7-1.3)	1.00
Prepared inside the home	67/448	(15)	209/1283	(16)	0.9	(0.6-1.2)	1.00
Prepared outside the home	25/432	(6)	46/1277	(4)	1.5	(0.8-2.6)	1.00
Shellfish	25/431	(6)	40/1274	(3)	2.2	(1.2-4.0)	0.23
Prepared inside the home	6/426	(1)	26/1275	(2)	0.8	(0.3-2.0)	1.00
Prepared outside the home	20/433	(5)	21/1273	(2)	3.1	(1.5-6.4)	0.08
Shrimp	73/441	(17)	174/1279	(14)	1.3	(0.9-1.9)	1.00
Prepared inside the home	37/435	(9)	134/1280	(10)	0.9	(0.6-1.3)	1.00
Prepared outside the home	38/432	(9)	63/1277	(5)	2.2	(1.3-3.5)	0.08
Steak	89/432	(21)	304/1274	(24)	0.9	(0.7-1.3)	1.00
Prepared at a table service restaurant	24/443	(5)	54/1277	(4)	1.3	(0.7-2.3)	1.00
Summer sausage	24/435	(6)	76/1270	(6)	0.9	(0.5-1.6)	1.00
Prepared inside the home	21/444	(5)	62/1272	(5)	0.9	(0.5-1.6)	1.00
Prepared outside the home	2/429	(0)	19/1266	(2)	0.2	(0-0.9)	0.55
Turkey	119/442	(27)	306/1278	(24)	1.3	(0.9-1.7)	1.00
Prepared inside the home	103/436	(24)	281/1284	(22)	1.2	(0.9-1.5)	1.00
Prepared outside the home	29/437	(7)	59/1275	(5)	1.3	(0.8-2.1)	1.00
Veal	3/435	(1)	7/1265	(1)	1.7	(0.4-7.1)	1.00
Prepared inside the home	2/428	(0)	6/1263	(0)	1.4	(0.2-7.1)	1.00
Prepared outside the home	1/432	(0)	2/1265	(0)	2.2	(0.2-22.5)	1.00
Venison	29/435	(7)	58/1270	(5)	1.4	(0.8-2.4)	1.00
Prepared inside the home	29/426	(7)	58/1267	(5)	1.5	(0.9-2.4)	1.00
Prepared outside the home	2/432	(0)	3/1269	(0)	2.6	(0.3-17.6)	1.00
Environmental							
Camping	32/441	(7)	31/1268	(2)	3.2	(1.8-5.7)	0.006
Compost in past 12 months	19/418	(5)	37/1234	(3)	1.7	(0.9-3.2)	1.00
Contact with animal-based dog treats (e.g., rawhides, pig's ear)	42/419	(10)	113/1231	(9)	1.0	(0.6-1.5)	1.00
Contact with animals	327/437	(75)	895/1261	(71)	1.2	(0.9-1.6)	1.00
Contact with any wild animals or their droppings	34/412	(8)	58/1215	(5)	1.7	(1.0-2.7)	0.84
Contact with bird feed	9/437	(2)	18/1256	(1)	1.5	(0.6-3.6)	1.00
Contact with birds	16/438	(4)	30/1257	(2)	1.2	(0.6-2.5)	1.00
Contact with cat food	72/443	(16)	243/1254	(19)	0.7	(0.5-1.0)	1.00
Contact with cats	131/438	(30)	374/1258	(30)	0.9	(0.7-1.2)	1.00
Contact with chicken feed	12/431	(3)	8/1255	(1)	5.1	(1.9-14.2)	0.06
Contact with chickens	15/433	(3)	19/1256	(2)	2.1	(0.9-4.7)	1.00
Contact with dog food	157/441	(36)	493/1250	(39)	0.8	(0.6-1.1)	1.00
Contact with dogs	271/438	(62)	727/1264	(58)	1.3	(1.0-1.6)	1.00
Contact with fish	23/437	(5)	82/1252	(7)	0.8	(0.5-1.3)	1.00
Contact with fish food	16/442	(4)	52/1255	(4)	0.9	(0.5-1.7)	1.00
Contact with goats	9/438	(2)	3/1255	(0)	21.2	(4.3-145.7)	0.005
Contact with reptile or amphibian food	5/438	(1)	17/1257	(1)	0.5	(0.2-1.5)	1.00
Contact with reptiles or amphibians	19/436	(4)	31/1255	(2)	1.5	(0.8-2.9)	1.00
Contact with wild animals other than deer and elk or their droppings	18/407	(4)	39/1219	(3)	1.5	(0.8-2.9)	1.00
Contact with wild deer or elk or their droppings	20/406	(5)	13/1214	(1)	4.7	(2.2-10.4)	0.006
Household member visited/worked on farm with animals	43/435	(10)	49/1254	(4)	3.5	(2.1-5.9)	<0.001
Household member visited/worked on farm with cows	22/431	(5)	19/1248	(2)	5.1	(2.4-11.3)	0.002
Household member visited/worked on farm with sheep	7/433	(2)	8/1247	(1)	4.2	(1.1-15.8)	0.6
Live on a farm	28/444	(6)	23/1277	(2)	5.6	(2.6-12.2)	<0.001
And have contact with calves feed	2/450	(0)	1/1271	(0)	4.2	(0.4-60)	1.00
And have contact with cattle feed	1/447	(0)	2/1269	(0)	0.7	(0-8.3)	1.00
And have contact with cattle living area or manure	4/447	(1)	2/1269	(0)	7.5	(1.1-75.1)	0.68
And have contact with cows	5/450	(1)	4/1263	(0)	6.2	(1.2-36.2)	0.49
With calves present	6/452	(1)	5/1272	(0)	5.4	(1.3-25.7)	0.43
With chickens present	11/443	(2)	2/1274	(0)	28.1	(6.5-178.6)	<0.001
With cows present	8/447	(2)	16/1273	(1)	1.4	(0.5-3.5)	1.00
With horses present	9/447	(2)	7/1278	(1)	3.2	(1.0-10.5)	0.74
With pigs present	3/443	(1)	0/1274	(0)	66.9	(4.7-9270.2)	0.05
With sheep present	3/442	(1)	1/1277	(0)	3.6	(0.4-42.9)	1.00
Live on, visit, or work on a farm, petting zoo, or fair	72/430	(17)	61/1258	(5)	8.0	(4.7-14.1)	<0.001
And have contact with calves	9/430	(2)	4/1242	(0)	11.8	(2.9-59)	0.02
And have contact with chickens	17/428	(4)	19/1241	(2)	2.9	(1.3-6.5)	0.22
And have contact with cows	13/435	(3)	7/1244	(1)	8.7	(2.7-32.9)	0.01
And have contact with cows or calves	19/423	(4)	6/1246	(0)	18.8	(5.8-70.6)	<0.001
With calves present	21/424	(5)	9/1250	(1)	23.3	(7.4-88.9)	<0.001
With chickens present	27/430	(6)	9/1255	(1)	26.1	(9.1-87.2)	<0.001
With cows or calves present	42/426	(10)	29/1258	(2)	9.3	(4.7-19.2)	<0.001

Characteristic or exposure*	Cases† (n=774) n/N†	(%)	Controls† (n=2,365) n/N†	(%)	Multivariable analysis OR	(95% CI)	P ‡
With cows present	40/429	(9)	29/1257	(2)	6.8	(3.5-13.5)	<0.001
With deer present	3/432	(1)	2/1249	(0)	4.1	(0.6-30.4)	1.00
With goats present	21/435	(5)	8/1250	(1)	15.7	(5.3-52.8)	<0.001
With horses present	22/429	(5)	17/1256	(1)	3.9	(1.6-9.6)	0.07
With pigs present	12/429	(3)	6/1252	(0)	13.6	(3.5-65)	0.008
With sheep present	14/430	(3)	7/1249	(1)	13.2	(3.9-51.4)	0.002
With turkeys present	2/428	(0)	4/1254	(0)	0.4	(0-3.2)	1.00
Use animal manure in garden in past 12 months	13/418	(3)	19/1227	(2)	2.5	(1.1-5.6)	0.48
Use compost or animal manure in garden in past 12 months	28/425	(7)	41/1233	(3)	2.5	(1.4-4.5)	0.09
Use prepackaged compost or manure in garden in past 12 months	15/420	(4)	29/1232	(2)	1.8	(0.9-3.6)	1.00
Visit a fair	11/439	(3)	11/1272	(1)	3.8	(0.9-15.3)	0.93
Visit a farm	40/434	(9)	25/1271	(2)	9.0	(4.6-17.9)	<0.001
And have contact with cattle feed	6/431	(1)	1/1262	(0)	12.9	(2.3-98.9)	0.1
And have contact with chicken living area or manure	3/431	(1)	1/1268	(0)	14.5	(1.8-165.9)	0.26
And have contact with cow living area or manure	7/431	(2)	4/1263	(0)	5.4	(1.3-24.1)	0.37
And have contact with horse feed	5/438	(1)	2/1269	(0)	20.9	(3.6-185.6)	0.03
And have contact with horse living area or manure	6/439	(1)	4/1270	(0)	7.5	(1.8-34)	0.15
With calves present	10/436	(2)	5/1264	(0)	9.4	(2.6-38.9)	0.03
With chickens present	12/430	(3)	5/1270	(0)	10.0	(3.4-31.2)	0.002
With cows present	21/436	(5)	9/1262	(1)	7.7	(3.2-19.5)	<0.001
With horses present	12/434	(3)	5/1269	(0)	12.8	(4-46.6)	<0.001
With sheep present	7/427	(2)	1/1262	(0)	19.7	(3.7-138.7)	0.02
With turkeys present	0/434	(0)	2/1266	(0)	0.1	(0-3.0)	1.00
Visit a petting zoo	7/440	(2)	9/1270	(1)	1.6	(0.4-5.5)	1.00
With cows present	3/451	(1)	3/1262	(0)	4.0	(0.5-34.5)	1.00
With goats present	3/433	(1)	5/1266	(0)	1.7	(0.3-8.5)	1.00
With horses present	1/437	(0)	2/1267	(0)	1.0	(0.1-11.7)	1.00
Work resulted in contact with live animals or carcasses (e.g., veterinarian, food production)	4/429	(1)	7/1258	(1)	2.0	(0.5-7.4)	1.00
Work, play, or help in garden	75/428	(18)	189/1241	(15)	1.3	(0.9-1.8)	1.00
Water							
Cattle near well at home	5/423	(1)	3/1254	(0)	4.2	(0.8-24.9)	1.00
Drink bottled water	308/414	(74)	887/1224	(72)	1.2	(0.9-1.5)	1.00
Drink untreated water (e.g., lake, spring, or river)	18/434	(4)	9/1252	(1)	6.6	(2.8-16.1)	0.001
Swallow water	-	-	-	-	-	-	-
At water park	9/432	(2)	9/1250	(1)	4.6	(1.5-14.1)	0.19
From lake	30/433	(7)	35/1238	(3)	3.0	(1.7-5.3)	0.02
From ocean	5/433	(1)	11/1252	(1)	1.5	(0.4-4.9)	1.00
From pool	52/427	(12)	168/1257	(13)	0.9	(0.6-1.3)	1.00
From splash pad or fountain	4/433	(1)	13/1247	(1)	0.8	(0.2-2.5)	1.00
From wading pool	8/425	(2)	20/1245	(2)	1.1	(0.4-2.6)	1.00
Swim or play	-	-	-	-	-	-	-
In irrigation ditch	3/433	(1)	1/1254	(0)	6.1	(0.6-87.1)	1.00
In lake, river, or stream	41/432	(9)	53/1249	(4)	2.5	(1.5-4.2)	0.02
In lake, river, or stream with nearby cattle	2/424	(0)	0/1237	(0)	13.4	(0.5-1277.8)	1.00
In ocean	7/436	(2)	15/1253	(1)	1.5	(0.5-4.0)	1.00
In pool	64/442	(14)	186/1257	(15)	0.8	(0.6-1.2)	1.00
In splash pad or fountain	7/431	(2)	18/1251	(1)	1.0	(0.4-2.4)	1.00
In wading pool	11/437	(3)	33/1251	(3)	0.8	(0.4-1.8)	1.00
In water	112/438	(26)	234/1253	(19)	1.5	(1.1-2.1)	0.33
In water park	12/436	(3)	10/1251	(1)	4.2	(1.5-11.7)	0.15
Treatment for well water at home other than with a whole-house point-of-entry device	15/413	(4)	55/1236	(4)	0.7	(0.3-1.2)	1.00
Use municipal water at home	334/446	(75)	963/1267	(76)	1.0	(0.7-1.3)	1.00
Use municipal water away from home	225/395	(57)	661/1167	(57)	1.1	(0.8-1.4)	1.00
Use private well water at home	76/429	(18)	193/1267	(15)	1.2	(0.8-1.7)	1.00
Use private well water away from home	29/366	(8)	65/1067	(6)	1.3	(0.7-2.1)	1.00
Use spring water at home	2/436	(0)	9/1246	(1)	1.4	(0.2-5.4)	1.00
Use spring water away from home	3/358	(1)	11/1045	(1)	0.8	(0.2-3.0)	1.00
Whole house point-of-entry treatment for well water at home	19/413	(5)	51/1229	(4)	1.5	(0.8-2.7)	1.00

*In the 7 days before illness began unless otherwise specified. Only exposures with sufficient sample sizes for analysis after nearest-neighbors matching are listed. Interviewers told respondents to consider foods prepared at any home to be prepared at home and foods prepared at a restaurant or commercial food service establishment to be prepared outside the home.
†The initial sample for each exposure was 774 non-infant non-traveler cases and 2,365 non-infant non-traveler controls. During nearest-neighbors matching, cases and controls without a match were excluded for the exposure under consideration. Thus, the numbers (i.e., N in the table) of cases and controls that were matched and included in the analysis for each exposure is smaller than the total. Number of exposed is denoted by n.
‡P is adjusted for multiple testing using the Benjamini-Hochberg-Yekutieli method
